# Sodium–glucose cotransporter-2 inhibitor alleviated atrial remodeling in STZ-induced diabetic rats by targeting TLR4 pathway

**DOI:** 10.3389/fcvm.2022.908037

**Published:** 2022-09-06

**Authors:** Xiaoping Zhan, Lijun Cheng, Ning Huo, Lin Yu, Changle Liu, Tong Liu, Guangping Li, Huaying Fu

**Affiliations:** Tianjin Key Laboratory of Ionic-Molecular Function of Cardiovascular Disease, Department of Cardiology, Tianjin Institute of Cardiology, The Second Hospital of Tianjin Medical University, Tianjin, China

**Keywords:** atrial remodeling, atrial fibrillation, dapagliflozin, diabetes mellitus, TLR4

## Abstract

**Purpose:**

The mechanism of sodium–glucose cotransporter-2 inhibitor (SGLT-2i) reducing the incidence of atrial fibrillation remains unclear. We hypothesize that sodium–glucose cotransporter-2 inhibitor alleviated atrial remodeling in STZ-induced diabetic rats by targeting TLR4 pathway.

**Methods:**

A total of 42 rats were randomly assigned into three groups: control group (CON group); diabetes group (DM group): diabetes mellitus rats were established by 65 mg/kg streptozotocin (STZ) intraperitoneal injection; and diabetes + dapagliflozin group (DM + DAPA group): diabetic rats were given DAPA gavage administration (DAPA 2mg/kg/d for 4 weeks by gavage administration), 14 rats in each group. Epicardial multiple-lead recording and intracardiac electrophysiology studies were performed to investigate the electrical remodeling in the heart and the atrial fibrillation inducibility in each group. Western blot analysis and real-time PCR were used to determine the protein and mRNA expression of toll-like receptor 4 (TLR4), interleukin receptor-associated kinase 1 (IRAK1), tumor necrosis factor receptor-associated factor 6 (TRAF6), nuclear factor-kappa B (NF-κB), and type I collagen (collagen I).

**Results:**

Compared with rats in CON group, rats in DM group showed marked myocardial fibrosis, ectopic pacing excitement, reduced conduction velocity, decreased cardiac function. TLR4/IRAK1/TRAF6/NF-κB, collagen I proteins expressions and incidence of atrial fibrillation (27.3%) were increased in DM group. Parts of these changes were reversed by treatment of DAPA. Incidence of atrial fibrillation was decreased in DM + DAPA group (2.8%).

**Conclusions:**

SGLT-2i dapagliflozin may prevent diabetic rats' atrial remodeling and reduce the inducibility of atrial fibrillation partly by targeting TLR4/IRAK1/TRAF6/NF-κB inflammatory pathway.

## Introduction

Atrial fibrillation (AF), one of the most common arrhythmias, is prone to major adverse cardiovascular events (1). Diabetes mellitus (DM) is an independent risk factor for atrial fibrillation (2). Sodium–glucose cotransporter-2 inhibitor (SGLT-2i) is applied in the treatment of symptomatic chronic heart failure (HFrEF) in adults with reduced ejection fraction, with or without type 2 diabetes mellitus (3). As a classical antidiabetic medication, it has become one of the novel cornerstones in the treatment of heart failure. There are clinical evidences that SGLT-2i reduces 32% hospitalization of heart failure and incidence of atrial fibrillation (4, 5). SGLT-2i can reduce the risk of cardiovascular events and all-cause mortality in patients with type 2 diabetes (6). However, the mechanism of SGLT-2i reducing the incidence of atrial fibrillation remains unclear. Recent research revealed that toll-like receptor 4 (TLR4) expression was upregulated under hyperglycemia (7). When activated TLR4 was silenced, it could inhibit atrial fibrosis and susceptibility to AF by regulating NLRP3-TGF-β in hypertensive rats (8).

Therefore, we hypothesize that SGLT-2i can alleviate atrial remodeling in STZ-induced diabetic rats by targeting TLR4 pathway.

## Materials and methods

### Rat model and study design

All procedures in this experiment were approved by the Animal Regional Ethics Committee of the Tianjin Medical University. A total of 42 healthy adult male Wistar rats (Beijing Huayu Kang Biotechnology, China), weighing 150–200 g, were housed in standard environmental conditions with food and water *ad libitum*. Rats were randomly assigned into three groups: control group (CON group), diabetes group (DM group) rats received a single intraperitoneal injection of 65 mg/kg streptozotocin (STZ) (STZ was dissolved in citrate buffer), and diabetes+dapagliflozin group (DM+DAPA group) diabetic rats received dapagliflozin (AstraZeneca Pharmaceuticals LP, United States) 2 mg/kg daily for 4 weeks by gavage. Diabetes mellitus models were validated by measuring blood glucose taken from the tail vein and defined as the blood glucose level higher than 11 mmol/L twice or 20 mmol/L once. Three groups of rats were kept together to ensure the same external environment such as temperature, humidity, and feeding as far as possible. Four weeks later, the animals in the three groups were subjected to blood glucose, blood pressure, epicardial multiple-lead recording, and intracardiac electrophysiological study, and molecular biology research was carried out after obtaining tissues.

### Blood pressure measurement

Rat was fixed with net and bag and then placed in a heating preservation tube to keep warm. Blood pressure was measured by using the tail-cuff method (BP98AL, Softron, Japan) as previously described (9). The measurements were repeated five times for each rat, and the average value was included.

### Histopathological studies

Histopathological studies were performed as described previously. Briefly, hearts were harvested and fixed in 10% formalin for at least 3 days. Tissues were cut transversely into 4–5 μm slices. Hematoxylin and eosin staining (Solarbio, Beijing, China) and modified Masson's trichrome staining (Solarbio, Beijing, China) were performed according to instructions to observe cell morphology and fibrotic area. Image Pro 6.0 was used for the analysis of the results.

### Echocardiography

The rats were anesthetized with 1.5% isoflurane and assessed by a Vevo 2100 system (VisualSonics Vevo 2100, SONICS, Newtown, CT, United States). Parasternal LV long-axis view, short axis at the mid-papillary muscle level, and four-chamber view were recorded during three consecutive cardiac cycles. Fractional shortening (FS %) and ejection fraction (EF %) were calculated. All the results were repeated three times for subsequent analysis.

### Intracardiac electrophysiology study

The intracardiac electrophysiology study was performed as described previously (10). The rats were fixed on the operating table, and the neck skin was exposed. The surface ECG was connected. A 1.6F catheter (EPR-802, Millar Instruments, United States) was inserted into right jugular vein. Surface ECG and intracardiac ECG were displayed and recorded using a PowerLab data acquisition system. Baseline ECG waveform was recorded before a series of subsequent stimulations. Exogenous stimulations were performed by an external stimulator (STG-3008, AD instruments, Australia) and applied at the electrode where atrial waveform was most pronounced. Atrial burst stimulation was performed at S1S1 stimulation cycle lengths starting from 40 ms with 2 ms stepwise reduction down to 20 ms. The stimulation was repeated five times, and the interval of each recovery period was 1 min. Sinus cycle length (SCL), Wenckebach cycle length (WCL), sinus node recovery time (SNRT), and effective refractory period (ERP) were defined as previously described and recorded to analyze the changes in cardiac electrical function. Atrial fibrillation was defined as a rapid irregular atrial rhythm with irregular R-R intervals lasting at least 1 sec. The corrected SNRT (CSNRT) was expressed according to the variation of sinus cycle length [CSNRT = SNRT–sinus cycle length (SCL)].

### Epicardial mapping technique

The epicardial electrical conduction characteristics were performed by epicardial mapping technique as described previously (11). Rats were anesthetized, artificially ventilated, and subjected to middle thoracotomy. A 36-electrode microelectrode array (MEA, Multichannel Systems, Britain) was put on epicardial surface in the left and right atrium. The moment of the fastest decline on the descending branch of the single heartbeat waveform is defined as the exciting point. The atrial waves with uniform atrial conduction were selected to measure the atrial conduction velocity, and at least three consecutive atrial waves were selected and recorded to calculate the atrial conduction heterogeneity and conduction heterogeneity index. Each recording lasted 5 sec. All the measurements were analyzed by EMapScope 4.0 software (MappingLab Ltd., United Kingdom).

### Western blot analysis

The heart tissues were quickly collected and frozen in liquid nitrogen for further research. Then, heart tissues were lysed in ice-cold RIPA buffer with a 1% protease and phosphatase inhibitor cocktail. The protein concentration in the lysis buffer was determined by bicinchoninic acid (BCA) protein assay reagent kit (Thermo Scientific, United States), and 20 μg proteins were separated by SDS-PAGE (8% or 10%) and transferred to a polyvinylidene fluoride microporous membrane (Millipore, Burlington, MA). Subsequently, membranes were blocked with 5% skim milk or 1% BSA and incubated with specific primary antibodies for toll-like receptor 4 (TLR4) (Abcam, rabbit Ab, 1:300), interleukin receptor-associated kinase 1 (IRAK1) (Abcam, rabbit Ab, 1:1,000), tumor necrosis factor receptor-associated factor 6 (TRAF6) (Santa Cruz, mouse Ab, 1:500), nuclear factor-kappa B (NF-κB) (Cell Signaling Technology, rabbit mAb, 1:1,000), type I collagen (collagen I) (Bioss, rabbit Ab, 1:1,000), β-actin (Proteintech, mouse Ab, 1:4,000–5,000) at 4°C overnight and followed by incubation with appropriate peroxidase-conjugated secondary antibodies. ImageJ software (NIH) was used for quantitative analysis.

### Real-time PCR

Total RNA was isolated with Eastep® Super Total RNA Extraction Kit (Promega, Shanghai, China) from heart and quantified using NanoDrop (Thermo Fisher Scientific, United States). Then, 1 μg RNA was reverse transcribed using the Reverse Transcription Kit (GenePharma, Shanghai, China). The primers for targets are listed in Table 3. Subsequently, cDNA was applied to the ABI 7500 Real-Time PCR System (Applied Biosystems, United States). β-actin was used as an internal control. The obtained amplification data were analyzed by using the 2^–ΔΔCt^ method.

### Statistical analysis

All data were expressed as mean ± SD or median with an interquartile range. ANOVA was used to make comparisons between multiple groups, followed by Tukey's *post–hoc* analysis for comparisons between two groups. Non–parametric Kruskal–Wallis test was used to analyze the data that did not conform to normal distribution. Fisher's exact test was used for evaluating the incidence of AF. All data were analyzed using SPSS 19.0 and GraphPad Prism 8. *P* < 0.05 was considered statistically significant. All figures were completed by GraphPad Prism 8.

## Results

### Effects of DAPA on basic parameters and cardiac function in STZ-induced diabetic rats

**Figure 1:**
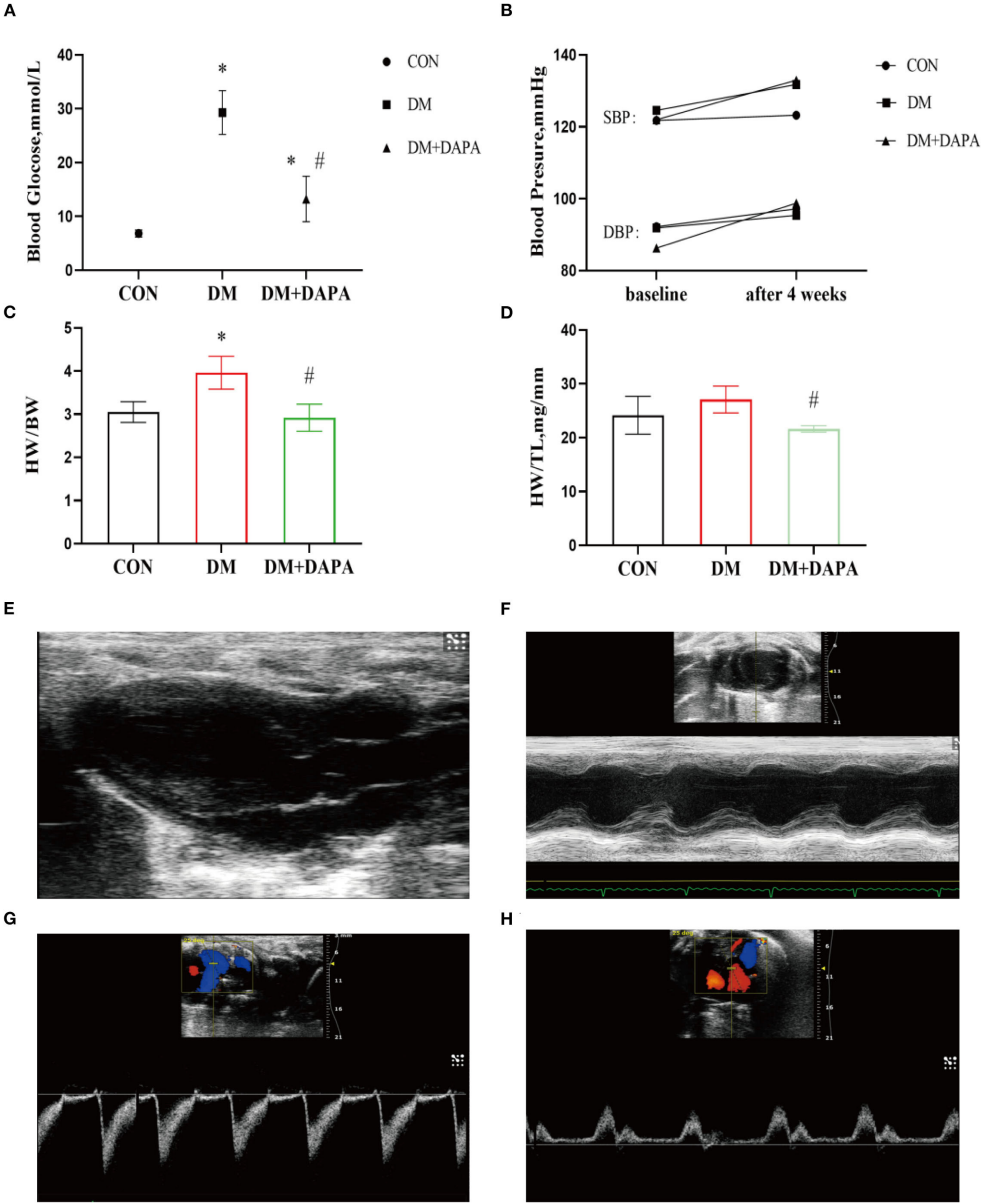
Effects of DAPA on the basic parameters and cardiac function in STZ-induced diabetic rats. **(A)** Random blood glucose after 4 weeks among three groups (*n* = 8); **(B)** systolic and diastolic blood pressure in baseline and after 4 weeks (*n* = 7); **(C)** heart weight to body weight (HW/BW) ratio in different groups (*n* = 8); **(D)** heart weight to tibia length (HW/TL) ratio in different groups (*n* = 8); **(E)** parasternal left ventricle long-axis view; **(F)** M-type echocardiogram of short-axis view; **(G)** Doppler flow imaging of short-axis view of the heart base; **(H)** Doppler flow imaging of four-chamber view. CON, control group; DM, diabetes group; DM + DAPA, diabetes + dapagliflozin group; SBP, systolic blood pressure; DBP, diastolic blood pressure. Data are expressed as the mean ± SEM. ^*^P < 0.05 vs. CON group. ^#^*P* < 0.05 vs. DM group.

Compared with CON group, the DM group showed remarkably an increase of blood glucose, and the blood glucose level was decreased in DM + DAPA group (6.838 ± 0.6567, 29.28 ± 4.074, and 13.23 ± 4.210, respectively, *P* < 0.0001, Figure 1A).

There was no significant difference in blood pressure among groups (shown in Figure 1B). Furthermore, the DM group rats tended to develop heart hypertrophy and decreased in DM + DAPA group as shown in Figures 1C,D.

Typical echocardiographic images are shown in Figures 1E–H. Left atrial diameter in DM group and DM + DAPA group was larger than in CON group, but there was no statistical difference (*P* > 0.05). Interventricular septum in DM rats was thinner than CON rats (*P* < 0.05), and these changes did not reverse in DM + DAPA group (*P* > 0.05). Left ventricular ejection fraction (LVEF) and left ventricular fractional shortening (LVFS) were decreased in DM rats (*P* < 0.05), and these changes were reversed by the administration of DAPA (shown in Table 1).

**Table 1:** Echocardiographic parameters.

	**CON**	**DM**	**DM + DAPA**	***P* values**
	**(*n* = 8)**	**(*n* = 8)**	**(*n* = 8)**	
LAD	4.011 ± 0.3483	4.571 ± 0.9002	4.723 ± 0.4867	0.1107
PAT	32.96 ± 8.505	33.11 ± 6.505	29.28 ± 7.753	0.5608
IVS	2.026 ± 0.2486	1.689 ± 0.2380*	1.689 ± 0.1020*	0.0155
LVID;d	6.439 ± 0.3950	7.185 ± 0.7655	6.508 ± 0.6074	0.0521
LVID;s	3.455 ± 0.6751	4.202 ± 0.5355*	3.348 ± 0.5044^#^	0.0152
LVPW	2.189 ± 0.3152	1.804 ± 0.1974	2.086 ± 0.5004	0.0864
EF (%)	78.19 ± 7.996	69.55 ± 3.389*	78.30 ± 6.495^#^	0.0260
FS (%)	50.21 ± 7.617	40.36 ± 2.755*	48.47 ± 6.918^#^	0.0171

LAD, anteroposterior diameter of left atrium; PAT, pulmonary artery acceleration time; IVS, interventricular septum; LVID;d, left ventricular internal diameter at diastolic period; LVID;s, left ventricular internal diameter at systolic period; LVPW, left ventricular posterior wall; EF, ejection fraction; FS, fractional shortening; CON, control group; DM, diabetes group; DM + DAPA, diabetes + dapagliflozin group. Data are expressed as the mean ± SEM. ^*^P < 0.05 vs. CON group. ^#^*P* < 0.05 vs. DM group.

### DAPA alleviates atrial pathological structure in diabetic rats

**Figure 2:**
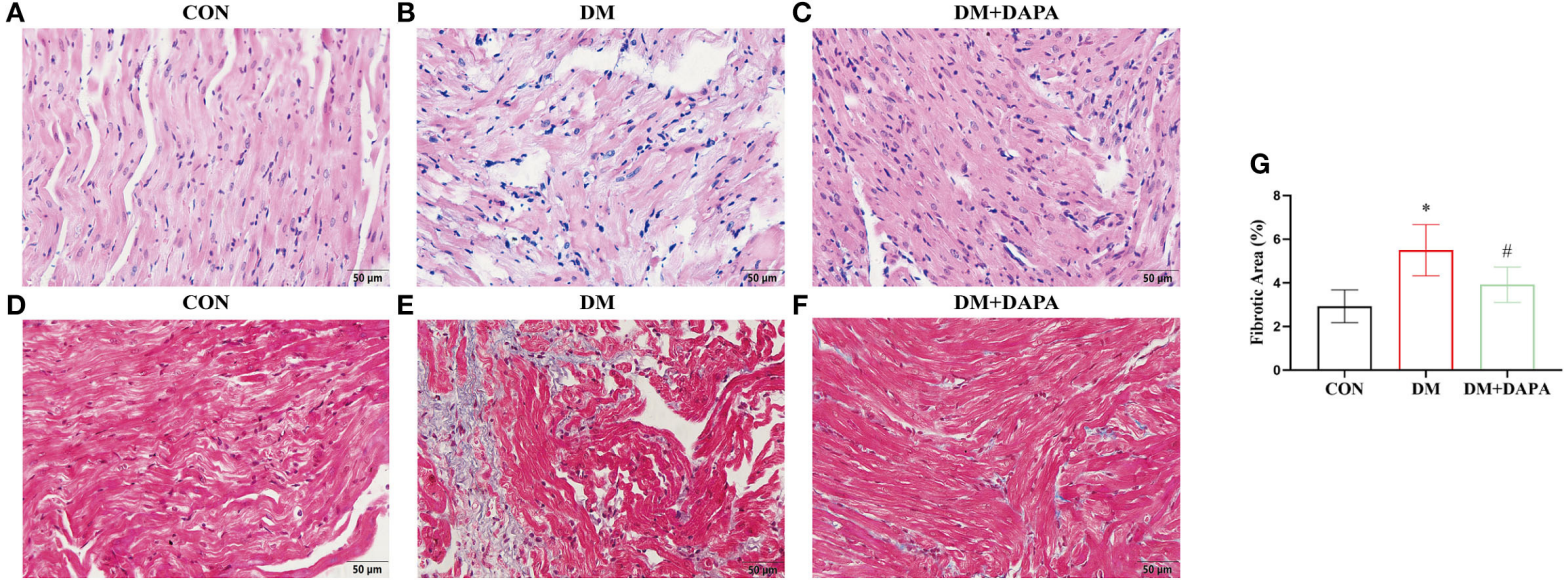
DAPA alleviates atrial remodeling in diabetic rats. **(A—C)** Typical images of H&E staining (n = 5); **(D–F)** representative pictures of Masson staining; **(G)** statistical analysis of Masson staining (n = 8). **(A,D)** CON group; **(B,E)** DM group; **(C,F)** DM + DAPA group; CON, control group; DM, diabetes group; DM + DAPA, diabetes + dapagliflozin group. Data are expressed as the mean ± SEM. ^*^P < 0.05 vs. CON group. #*P* < 0.05 vs. DM group.

H&E staining is shown in Figures 2A–C. The cell arrangement was disorder, and the cross-sectional areas of atrial cardiomyocytes were increased in DM rats and reversed in DM + DAPA group. Fibrotic area was higher in DM group compared with CON group, and fibrotic area was decreased in DM + DAPA group (2.933 ± 0.7480 vs. 5.502 ± 1.174 vs. 3.923 ± 0.8100, *P* = 0.0008, respectively, Figures 2D–G).

### Effects of DAPA on epicardial electrical conduction characteristics in STZ-induced diabetic rats

**Figure 3:**
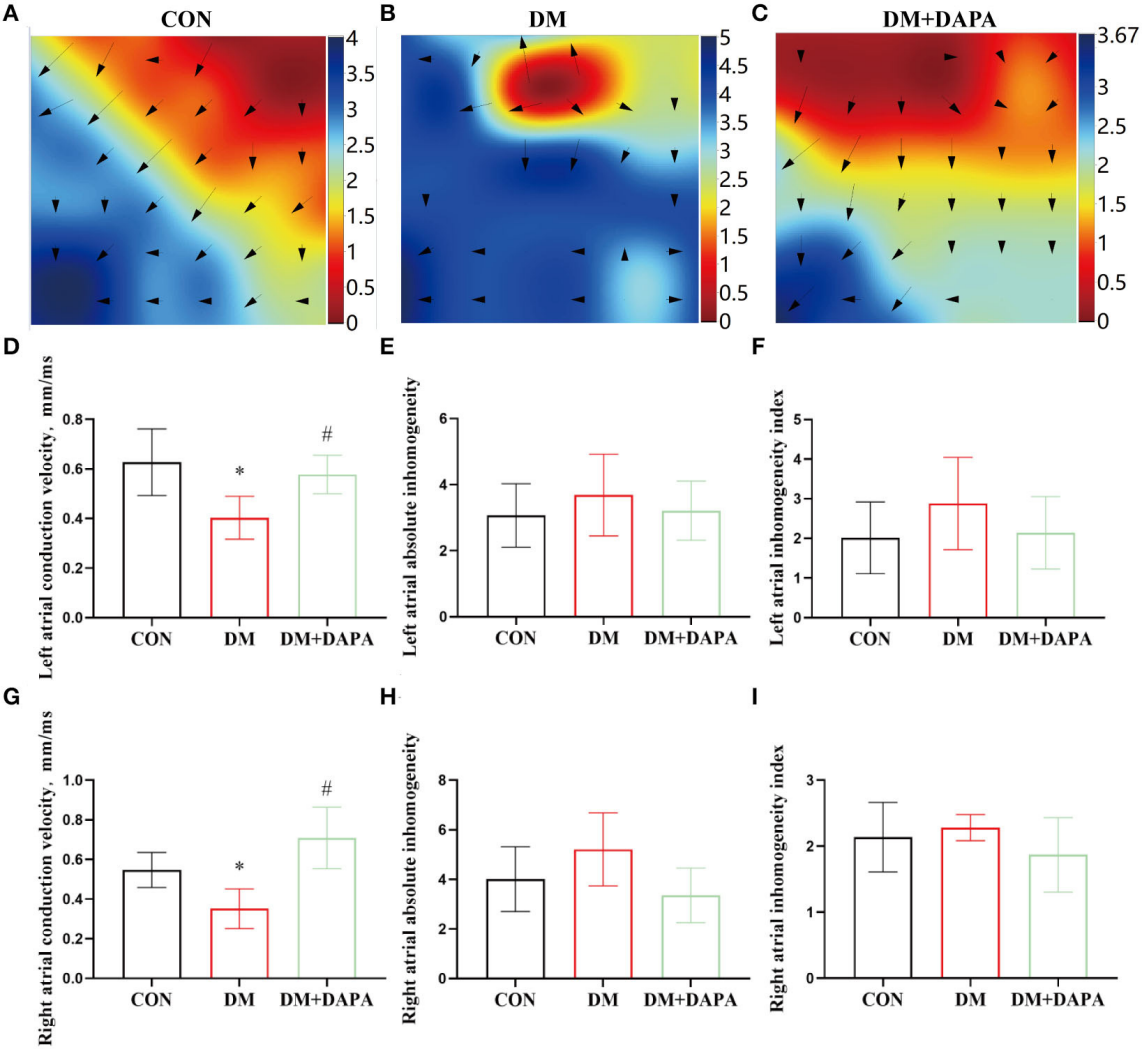
Effects of DAPA on epicardial electrical conduction characteristics in STZ-induced diabetic rats. **(A–C)** Typical images of electrical mapping; The arrow represents the direction of the excitement; **(D)** statistical analysis of conduction velocity in left atrium (*n* = 8); **(E)** statistical analysis of absolute inhomogeneity in left atrium (*n* = 8); **(F)** statistical analysis of inhomogeneity index in left atrium (*n* = 8); **(G)** statistical analysis of conduction velocity in right atrium (*n* = 8); **(H)** statistical analysis of absolute inhomogeneity in right atrium (*n* = 8); **(I)** statistical analysis of inhomogeneity index in right atrium (*n* = 8). CON, control group; DM, diabetes group; DM + DAPA, diabetes + dapagliflozin group. Data are expressed as the mean ± SEM. *P < 0.05 vs. CON group. ^#^*P* < 0.05 vs. DM group.

Epicardial electrical conductivity was measured by epicardial mapping technique. The representative epicardial mapping images are presented in Figures 3A–C. In the CON group, electrical conduction of atrium was uniform and spread away into the surrounding area. There were uneven conductions in DM rats, which were partly reversed in DM + DAPA group. Left atrial conduction velocity was decreased in the DM group compared with the CON group and increased in DM + DAPA rats (0.6274 ± 0.1342, 0.4030 ± 0.08665, and 0.5775 ± 0.07739, *P* = 0.0029, Figure 3D). Compared with CON group, left atrial conduction dispersion was greater in DM group but there was no significance, and DPAP administration could not change the higher dispersion (absolute inhomogeneity: 3.069 ± 0.9607 vs. 3.687 ± 1.237 vs. 3.213 ± 0.8930, *P* > 0.05; index: 2.015 ± 0.9049 vs. 2.880 ± 1.166 vs. 2.143 ± 0.9149, *P* > 0.05, Figures 3E,F). Right atrial conduction velocity was decreased in DM group and reversed in DM + DAPA rats (Figure 3G). Higher right atrial conduction dispersion (Figures 3H,I) was observed in DM rats and DM + DAPA group compared with CON group.

### DAPA could inhibit atrial electrical remodeling and the occurrence of atrial fibrillation in diabetic rats

**Figure 4:**
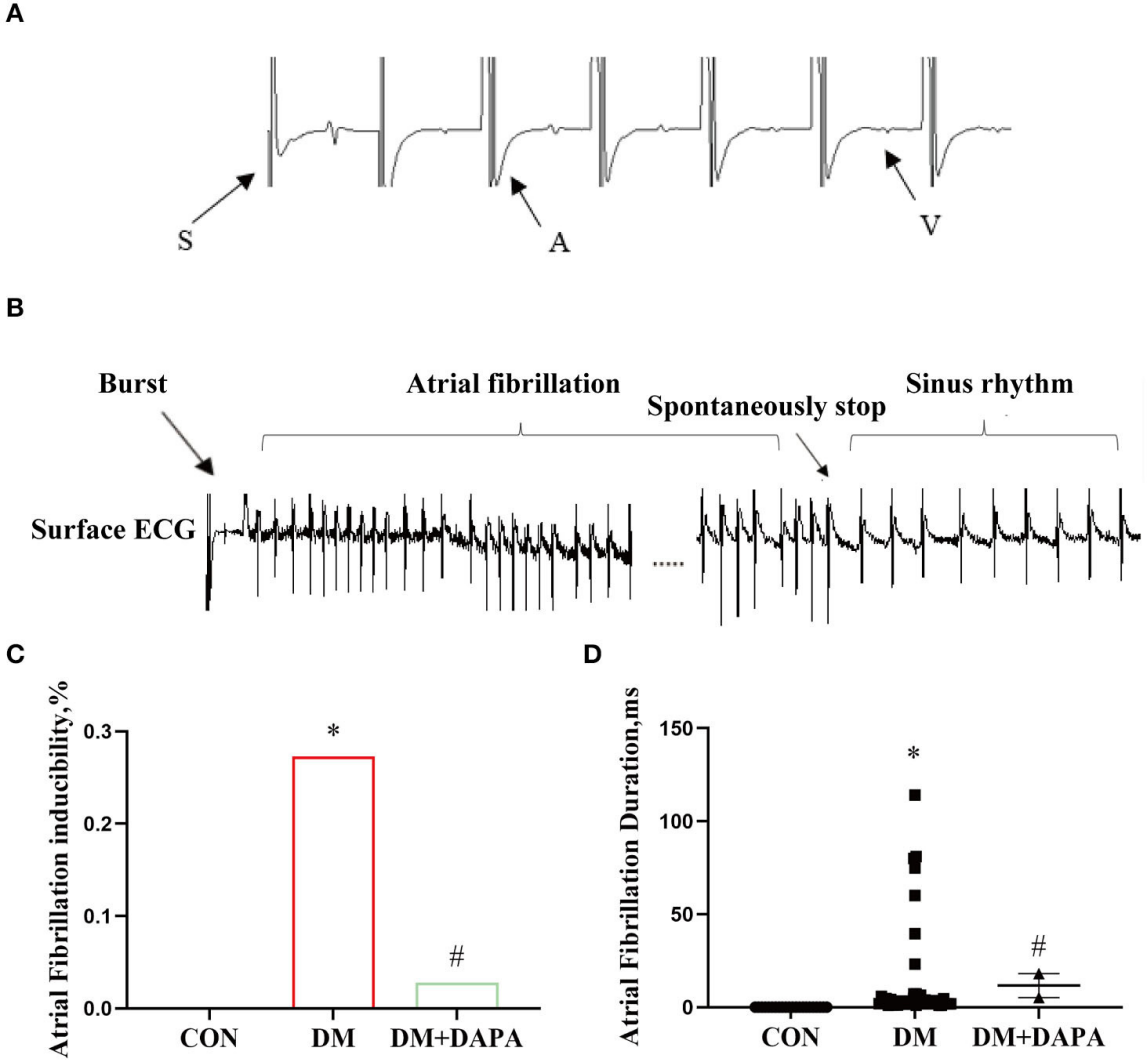
DAPA could inhibit atrial electrical remodeling and the occurrence of atrial fibrillation in diabetic rats. **(A)** Large stimulation artifacts are recorded on the surface ECG, following atrial wave and ventricular; **(B)** atrial fibrillation spontaneously stops after a series of burst; **(C)** atrial fibrillation inducibility; **(D)** statistical analysis of atrial fibrillation duration. CON, control group; DM, diabetes group; DM + DAPA, diabetes + dapagliflozin group. Data are expressed as the mean ± SEM. *P < 0.05 vs. CON group. ^#^*P* < 0.05 vs. DM group. The arrow represents the direction of the excitement.

In order to further confirm the effect of DAPA on atrial electrical remodeling in diabetic rats, we performed intracardiac electrophysiology study. During intracardiac stimulation, large stimulation artifacts are recorded on the surface ECG, following atrial wave and ventricular wave as shown in Figure 4A. Figure 4B represents atrial fibrillation after a series of burst stimulations and subsequently spontaneous converting to sinus rhythm. As shown in Figure 4C, the same exogenous stimulations were applied in three groups, and the inducibility of atrial fibrillation in DM group was 27.3%, compared with the control group 0% (*P* < 0.0001). The occurrence of atrial fibrillation was lower in DM + DAPA rats (2.8%) than in diabetic rats (*P* < 0.0001). Meanwhile, atrial duration was counted, and the duration of atrial fibrillation in DM rats was 1.05 s to 114.045 s, while the duration of AF in DM + DAPA rats varied from 5.18 s to 18.07 s (*P* < 0.05, Figure 4D). As shown in Table 2, SCL in DM rat was significantly prolonged compared with that in the control group (*P* < 0.0001, Table 2). There was marked prolongation in WCL, SNRT, CSNRT, and ERP in DM rats than in CON rats as shown in Table 2 (*P* < 0.05). Meanwhile, SCL, SNRT, CSNRT, and ERP could be abbreviated in DM + DAPA group (*P* < 0.05, Table 2), and there is no difference in WCL between DM rats and DM + DAPA rats (*P* > 0.05, Table 2).

**Table 2:** Electrophysiological parameters.

	**CON**	**DM**	**DM+DAPA**	***P* values**
	**(*n* = 9)**	**(*n* = 10)**	**(*n* = 7)**	
Weight, g	397.9 ± 59.50	211.5 ± 34.55*	298.1 ± 27.26*	<0.0001
SCL, ms	149.6 ± 16.58	190.8 ± 15.30*	152.0 ± 14.34^#^	<0.0001
Wenckebach cycle length, ms	87.78 ± 10.34	104.5 ± 14.50*	92.57 ± 12.70	0.0230
SNRT, ms	184.0 ± 19.54	232.4 ± 28.00*	180.9 ± 19.73^#^	0.0004
CSNRT, ms	36.00 ± 6.414	47.33 ± 21.79	30.33 ± 7.840	0.0948
ERP, ms	74.22 ± 8.342	89.00 ± 6.728*	70.57 ± 5.893^#^	<0.0001

SCL, sinus cycle length; AV, atrioventricular; WCL, Wenckebach cycle length; SNRT sinus node recovery time; CSNRT, corrected sinus node recovery time; ERP, the effective refractory period. CON, control group; DM, diabetes group; DM + DAPA, diabetes + dapagliflozin group. Data are expressed as the mean ± SEM. ^*^P < 0.05 vs. CON group. ^#^*P* < 0.05 vs. DM group.

**Table 3:** Primer sequences.

**Primer sequences**		
**Gene**		**Primer sequence(5^′^–3^′^)**
TLR4	forward	CTGGCCTCATCTTCATTGT
	reverse	GGGCTTCTTGAGTCTTCT
IRAK1	forward	CTCTGCCTCCACCTTCCTC
	reverse	AACCACCCTCTCCAATCCT
TRAF6	forward	AAAGCGAGAGATTCTTTCCCTG
	reverse	ACTGGGGACAATTCACTAGAGC
NF- κb	forward	TCTGTTTCCCCTCATCTTT
	reverse	TGGTATCTGTGCTTCTCTC
Collagen I	forward	CCCAGCGGTGGTTATGACTT
	reverse	TCGATCCAGTACTCTCCGCT
β-actin	forward	CCGCCTTGGAGTCCATCTAC
	reverse	GCGGCTTGTACCACTCTCG

### DAPA inhibits TLR4/IRAK1/TRAF6/NF-κB pathways and collagen I expression in atrium

**Figure 5:**
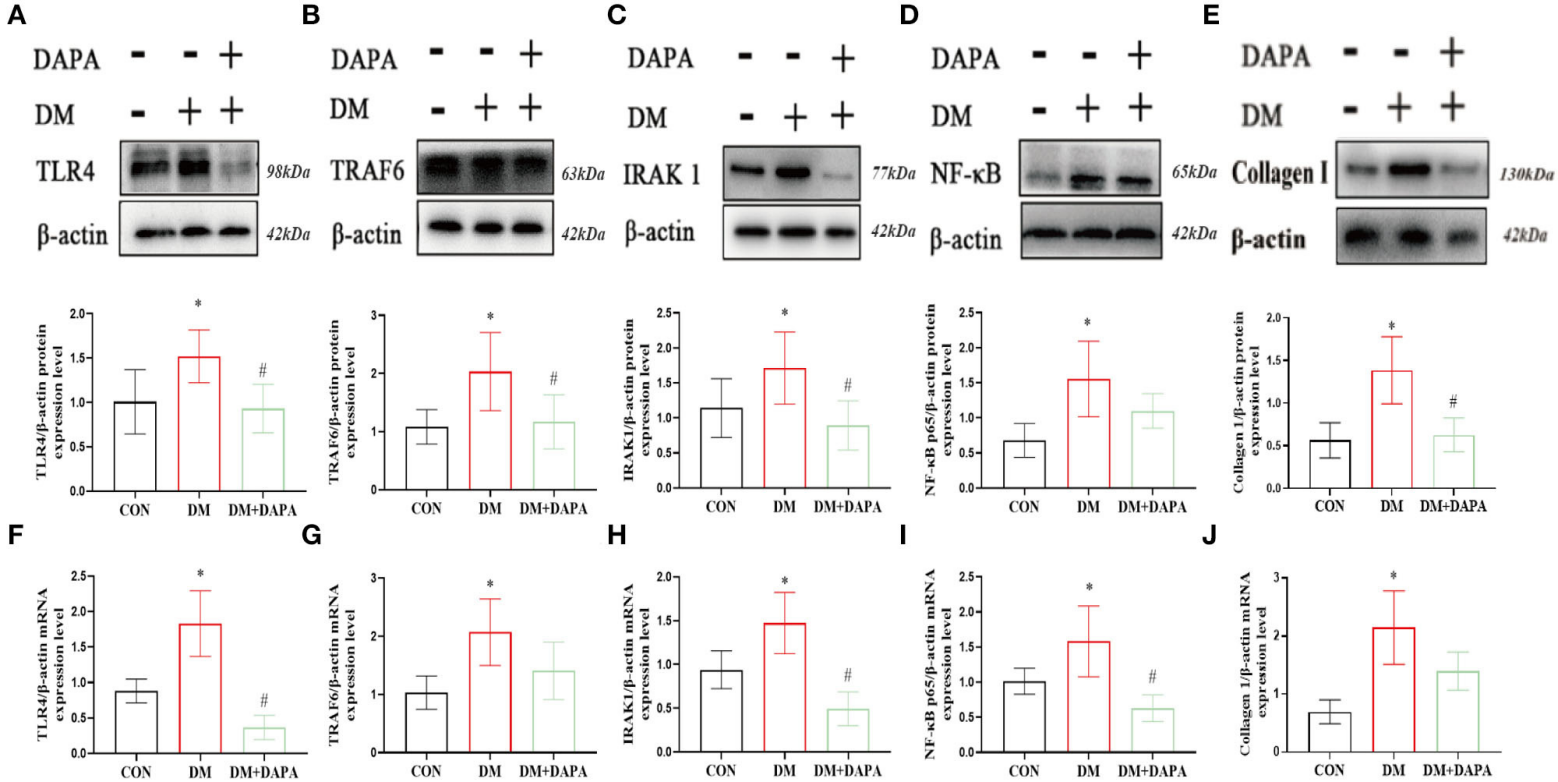
DAPA inhibits TLR4/IRAK1/TRAF6/NF-κB inflammatory pathways and Collagen I in atrium. **(A–E)** Protein expression of TLR4, TRAF6, IRAK1, NF-κB and Collagen I (*n* = 5-8). **(F–J)** mRNA expression of TLR4, TRAF6, IRAK1, NF-κB, and Collagen I (*n* = 5). CON, control group; DM, diabetes group; DM + DAPA, diabetes + dapagliflozin group. Data are expressed as the mean ± SEM. *P < 0.05 vs. CON group. #*P* < 0.05 vs. DM group.

Finally, in order to reveal the possible mechanism, we verified the role of TLR4/IRAK1/TRAF6/NF-κB in the inhibition of atrial remodeling and atrial fibrillation by DAPA. Compared with the CON group, TLR4, IRAK1, TRAF6, NF-κB, and collagen I were upregulated in diabetic rats, while suppressed in DM+DAPA group (TLR4: 1.008 ± 0.3615 vs. 1.519 ± 0.2976 vs. 0.9314 ± 0.2746, *P* = 0.0140; IRAK1: 1.141 ± 0.4182 vs. 1.712 ± 0.5146 vs. 0.8939 ± 0.3516, *P* = 0.0030; TRAF6: 1.083 ± 0.2963 vs. 2.033 ± 0.6714 vs. 1.169 ± 0.4642, *P* = 0.0029; NF-κB: 0.6789 ± 0.2403 vs. 1.552 ± 0.5397 vs. 1.099 ± 0.2456, *P* = 0.0003; collagen I: 0.5637 ± 0.2079 vs. 1.387 ± 0.3925 vs. 0.6279 ± 0.1973, *P* = 0.0015) (shown as in Figures 5A–E). As shown in Figures 5F–J, the mRNA expression of TLR4, IRAK1, TRAF6, NF-κB, and collagen I was increased in DM rats and downregulated in DM + DAPA group (TLR4: 0.8798 ± 0.1681 vs. 1.829 ± 0.4619 vs. 0.3664 ± 0.1717, *P* < 0.0001; IRAK1: 0.9381 ± 0.2163 vs. 1.472 ± 0.3509 vs. 0.4934 ± 0.1929, *P* = 0.0003; TRAF6: 1.032 ± 0.2877 vs. 2.071 ± 0.5725 vs. 1.407 ± 0.4928, *P* = 0.0151; NF-κB: 1.014 ± 0.1856 vs. 1.580 ± 0.5045 vs. 0.6292 ± 0.1899, *P* = 0.0017; collagen I: 0.6919 ± 0.2063 vs. 2.144 ± 0.6320 vs. 1.394 ± 0.3283, *P* = 0.0013).

## Discussion

This study indicated that there were structural remodeling and dysfunctions of the heart in diabetic rats, which let the DM rats prone to atrial fibrillation. Part of these changes can be alleviated by the administration of DAPA. DAPA may have cardiac protective benefits *via* inhibiting TLR4/IRAK1/TRAF6/NF-κB pathway.

### Atrial remodeling and diabetes mellitus

AF is a major arrhythmia in clinic, and its basic therapy includes rate control, rhythm control, and cerebral stroke. As is known to us, atrial fibrillation predisposes to worse prognosis in the context of diabetes mellitus (12). A persistent chronic inflammatory state is a hallmark of DM. Previous studies have indicated that the interaction between diabetes mellitus and atrial fibrillation is related to structural, electrical, electromechanical, and autonomic remodeling (12). Abnormal deposition and distribution of fibrosis are often closely linked to disruption of myocardial architecture (13). Evidences suggest that inflammation (14), oxidative stress (15), and mitochondrial dysfunction (16) were attributed to the fibrosis. The deposition of cardiac collagen fibers depends on the dynamic regulation process between metalloproteinases (MMPs) and tissue inhibitors of metalloproteinases (TIMPs) in cardiac tissue (17). However, in diabetes, the balance between MMP and TIMP is broken, resulting in excessive accumulation of collagen fibers (17). Suffering from ischemia, inflammation, and toxic insult, the normal myocardium was replaced with fibrous tissue, leading to structural remodeling (13). Echocardiography showed that interventricular septum in DM rats was thinner than CON rats. The situation may be due to ventricular dilatation and ventricular volume overload or the large body size difference between diabetic and normal rats (18). Therefore, we introduced standardized indexes of cardiac hypertrophy (e.g., HW/BW, HW/TL), excluding body size differences, and the results showed that the phenomenon of cardiac hypertrophy occurred in diabetic rats. The formation of fibrosis can interfere with the normal function of intercellular gap junctions and ion channels, decrease atrial effective refractory period (ERP) and action potential duration (APD), slower atrial conduction, and dysfunction of ion channels (19). Similar changes in electrical properties were verified in diabetes-induced atrial fibrillation (20). Therefore, the deposition of cardiac collagen fibers is regarded as the vitally important histological substrate of arrhythmias.

### TLR4/IRAK1/TRAF6/NF-κB pathway in atrial fibrillation

The TLR family, primarily recognized as a receptor that initiates innate immunity, is involved in the progression of tumorigenesis (21). Recent research found that compared with myocarditis, pericarditis may be a cause of atrial arrhythmias (22). TLR4 signaling participated in the pericardium–myocardium interactions inducing atrial arrhythmogenesis (23). Classically, lipopolysaccharide-induced activation of TLR4 results in the activation of a series of downstream inflammatory molecules, including IRAK1, TRAF6, NF-κB (24). Excessive activation of TLR4/IRAK1/TRAF6/NF-κB pathway commonly exists in myocardial inflammation (25). For example, IRAK1 participates in the heat shock protein 60 (HSP60)/TLR4 signaling, mediating myocardial apoptosis and inflammation after ischemia/reperfusion (I/R) shocking (24). Milano et al. (25) reported that TRAF6 is a contributor to the doxorubicin/trastuzumab-induced cardiac toxicity. NF-κB, composed of p50 (NF-κB1) or p52 (NF-κB2) usually associated with members of the Rel family (p65, c-Rel, Rel B), is reported to improve mitochondrial morphology and function (26) and directly affect the cardiomyocytes function (27). Previous researches are in line with our results, and TLR4/IRAK1/TRAF6/NF-κB pathway was markedly activated under hyperglycemia.

### SGLT-2i and atrial remodeling

SGLT-2 receptors are mainly distributed in the proximal convoluted tubules and are responsible for nearly 90% of glucose reabsorption (28). SGLT-2i is a new class of hypoglycemic agents that demonstrated excellent cardioprotective effects, reducing atherosclerotic events and protecting renal function (29). It significantly improved both cardiac diastolic and strain function, slightly lower body weight, blood pressure, and waist circumference (30). The cardioprotective effect associated with SGLT-2i was previously thought to be related to their diuretic and antihypertensive effects, which ameliorate ventricular loading (28). Unlike the loop diuretic bumetanide, SGLT-2i primarily reduced interstitial fluid without affecting blood volume (31). A meta-analysis showed that SGLT-2 inhibitors could not only reduce body weight and hematocrit but also significantly reduce blood pressure in patients with type 2 diabetes (32). The DAPA is a kind of SGLT-2i, which have little effect on SBP in patients with HFrEF (33). The results of this paper show that there is no significant difference between diabetic group and diabetic+DAPA group in SBP and DBP. These results suggest that the cardioprotective effect of DAPA may rely on other mechanisms. Under normoglycemic situation, DAPA attenuates reactive oxygen species (ROS) production and connexin 43 phosphorylation, reducing the occurrence of arrhythmia in infarcted rat (33). The mechanism of cardiac benefits of SGLT-2i may be independent of their hypoglycemic effects (34). Current studies support the use of SGLT-2i in the treatment of diseases other than diabetes mellitus. The results of this paper show that in addition to hypoglycemia, dapagliflozin may prevent diabetic rats' atrial remodeling and reduce the inducibility of atrial fibrillation. This is consistent with the results of previous studies that SGLT-2i can reduce the occurrence of atrial fibrillation (35).

DAPA treatment can attenuate electrical remodeling in AT II-stressed diabetic mice, an effect that was associated with inhibition of voltage-dependent L-type calcium channel (CACNA1C), the sodium–calcium exchanger (NCX), the sodium–hydrogen exchanger 1 (NHE) membrane transporters and fibrosis as well as inflammation (36). Cytoplasmic Na^+^ and Ca^2+^ concentration upregulates mitochondrial Ca^2+^ concentration, through affecting the activity of cardiac sodium-hydrogen exchanger (NHE), without concerning SGLT-2 receptor (36). In cardiomyocytes, NHE1 is the major exchanger isoform modulating sodium–proton exchange. The activity of NHE has been proved to be elevated in patients with severe heart failure and atrial fibrillation, suggesting that it may be associated with the pathogenesis of atrial fibrillation and heart failure (37). Uthman et al. revealed that SGLT-2i modulated myocardial fibrosis by inhibiting NHE1 activity, which reduced calcium influx into the myocardium and, consequently, mitochondrial damage (38). Moreover, SGLT-2i might modulate nutrient availability in cardiomyocytes and might influence the cardioprotective effect (39). The evidence also indicated that SGLT-2i exerts a cardioprotective effect by regulating energy metabolism and by activating autophagy when cells are in the starvation state following a decrease in the body glucose burden (39). SGLT-2 inhibitor can alleviate cardiac inflammation by regulating the macrophage polarization *via* STAT3 signaling and interfering with oxidative stress and glucotoxicity (40, 41).

### SGLT-2i application in anticancer-induced cardiotoxicity

The disorder of apoptotic mechanism is a pathogenic mechanism to inspire the onset of cancer. ped/pea-15, as a widely recognized antiapoptotic protein, has been found to be overexpressed in T2DM (42). At the same time, the overexpression of ped/pea-15 is associated with the increase of the susceptibility in chemically induced skin tumor development (43). Recent studies also have shown that diabetes is an important risk factor for colorectal cancer. Common systemic metabolic diseases, including obesity and diabetes, further modify the interplay between adipose tissue (AT) and breast cancer. Indeed, metabolic perturbations are accompanied by well-known alterations of AT functions, which might contribute to worsen cancer phenotype (44). As a common concomitant disease of diabetes, obesity can not only activate an inflammatory response, but also actively produce free fatty acids, adipokines, angiogenic factors, and extracellular matrix components as an endocrine organ, and ultimately build a microenvironment-supporting tumor.

Immune checkpoint inhibitors (ICIs) have revolutionized cancer treatment, achieving unprecedented efficacy in multiple malignancies (45). However, ICIs are associated with immune-related adverse events involving cardiotoxicity (46). Quagliariello V et al. took the first evidence that hyperglycemia exacerbates ipilimumab-induced cardiotoxicity and decreases its anticancer efficacy in MCF-7 and MDA-MB-231 cells. The study also sets the stage for further tests on other breast cancer cell lines and primary cardiomyocytes and for preclinical trials in mice aimed to decrease glucose through nutritional interventions or administration of gliflozines during treatment with ipilimumab (47).

Quagliariello V et al. aimed to evaluate the effects of SGLT-2i on myocardial strain of nondiabetic mice treated with doxorubicin (DOXO). They concluded that EMPA reduced ferroptosis, fibrosis, apoptosis, and inflammation in doxorubicin-treated mice through the involvement of NLRP3- and MyD88-related pathways, resulting in significant improvements in cardiac functions (48). The protective effects of SGLT-2i may also be due to its potent antioxidant properties, which protect cardiac tissue from oxidative damage and help to maintain myocardial cell membrane integrity and function (48). SGLT-2i can also ameliorate sunitinib-induced cardiac dysfunction by regulating AMPK–mTOR signaling pathway-mediated cardiomyocyte autophagy (49). Tian et al. found that DAPA could mitigate the cardiac fibrosis and inhibit the endothelial-tomesenchymal transition via AMPKα/TGF-β/Smad signaling (50).

### SGLT-2i and its renoprotection

A previous study reported that SGLT-2i provided renoprotection by lowering the intraglomerular hypertension by modulating the pre- and post-glomerular vascular tone (39). Diabetic rats show an upregulation of renal fibroblasts, mesangial cells, and podocytes as well as nicotinamide adenine dinucleotide phosphate oxidase (NOXs) and increased production of reactive oxygen species in renal tissues. Inhibition of NOXs significantly protects the kidney from structural and functional renal damage. This may be the molecular mechanism of Canagliflozin's effect on renal protection (51). SGLT-2i plays a renoprotective role in rats with acute kidney injury after myocardial infarction by increasing the circulating level of ketone body d-β-hydroxybutyrate (βOHB), and βOHB upregulates antioxidant molecules by inhibiting histone deacetylases (HDACs) (52). Panchapakesan et al. reported that empagliflozin could reduce the expression of TLR4 and the secretion of IL-6 and NF-κB in human renal proximal convoluted tubular epithelial cells. Thus, it reduces the expression of inflammatory factors and fibrotic markers induced by high glucose toxicity (53).

### SGLT-2i reduces the inducibility of AF *via* TLR4 pathway

The specific mechanism by which SGLT-2i can reduce the incidence of atrial fibrillation after DM is unclear. These changes may be related to the abnormal distribution of intercellular gap junction proteins (54), activation of reactive inflammatory signaling pathways (55), and ion channel dysfunction disorder (56). The protective effect of drugs on the heart is related to a variety of signal pathways. Previous studies have suggested that the cardioprotective effect of SGLT-2i may be related to the effect of inhibiting inflammatory pathways. SGLT-2i not only inhibits the inflammatory responses, reducing oxidative stress productions and mitochondrial stress, but also changes the electrical characteristics of the heart, affecting the function of ion channels, the disordering of electrical conduction. As mentioned earlier, the immune inflammatory response, especially TLR4, is consistently involved throughout diabetic vascular disease (56). TLR4 can induce and amplify the inflammatory response, and it plays an important role in cell proliferation, differentiation, and apoptosis (57). Previous studies have demonstrated that TLR4 can be involved in hyperinsulinemia, insulin resistance, lipid metabolism disorder, endothelial cell dysfunction, and blood coagulation (58). TLR4 could mediate the inflammatory response via activating the NF-κB pathway and downstream inflammatory factors to aggravate the damage of the inflammatory response (59). In this study, TLR4 and NF-κB proteins and collagen I protein expressions were significantly increased in DM rats, and they were downregulated in DAPA treatment group. The activation of NF-κB pathway and the subsequent overexpression of its downstream targets such as transforming growth factor-β1 (TGF-β1) are a critical pathway in progressive diabetic nephropathy (60).

In a diabetic rabbit model, we have previously shown that probucol prevented atrial remodeling and suppresses AF development effected on oxidative stress, NF-κB, TGF-β, and TNF-α overexpression (61). So, in this study, the DAPA treatment prevented atrial remodeling and suppresses AF development partly by suppressing the overexpression of the TLR4 and NF-κB involved in an immune inflammatory response.

Elaheh Abdollahi and his colleagues revealed that DAPA exerted direct anti-inflammatory effects, at least partly, by inhibiting the expression of TLR4 and activation of NF-κB along with the secretion of pro-inflammatory (62). However, experimental results in cell model should be proved in real organism. It is of significance to use animal model for the clinical transformation of drugs. Secondly, our manuscript revealed that SGLT-2i produces a marked cardioprotective effect through the TLR4/NF-κB pathway in diabetic animal model and SGLT-2i could suppress atrial structural remodeling and electrical remodeling and reduce the incidence of AF in diabetic rats. In conclusion, our present results show that the cardiac protection of DAPA may rely on the inhibition of TLR4/IRAK1/TRAF6/NF-κB pathway. However, the mechanism of DAPA for atrial fibrillation still needs validation in the future work.

### Study limitations

There exist several limitations that should be noted. Firstly, we did not verify the relationship between DAPA and TLR4/IRAK1/TRAF6/NF-κB pathway *in vitro*, and we could not further clarify that what mediators DAPA interacts with the TLR4 pathway. Secondly, we cannot rule out the effect of DAPA on control heart, which may cause disturbance of ion channel function and possibly have adverse effects on heart function. Thirdly, ion channel currents of DAPA administration were not studied in this study.

## Conclusions

SGLT-2i dapagliflozin may prevent diabetic rats' atrial remodeling and reduces the inducibility of atrial fibrillation. TLR4/IRAK1/TRAF6/NF-κB pathway is involved in this process.

## Data availability statement

The raw data supporting the conclusions of this article will be made available by the authors, without undue reservation.

## Ethics statement

The animal study was reviewed and approved by Animal Regional Ethics Committee of the Tianjin Medical University.

## Author contributions

HF conceived the work and designed the experiments. XZ and LC recorded the data and wrote the manuscript. NH and LY performed the statistical analysis. CL, TL, GL, and HF participated in the critical manuscript revision. All authors read and approved the final manuscript.

## Funding

This study was funded by Tianjin Natural Science Foundation (16JCYBJC25000, 21JCYBJC01740, and 21JCYBJC01460), Key Laboratory Scientific Research Foundation of Second Hospital of Tianjin Medical University (2018ZDSYS03 and 2019ZDSYS03), Clinical Study of Second Hospital of Tianjin Medical University (2019LC03), Tianjin Key Medical Discipline (Specialty) Construction Project, Tianjin Key Medical Discipline(Specialty) Construction Project (TJYXZDXK-029A), and National Natural Science Foundation of China (No. 82100342).

## Conflict of interest

The authors declare that the research was conducted in the absence of any commercial or financial relationships that could be construed as a potential conflict of interest.

## Publisher's note

All claims expressed in this article are solely those of the authors and do not necessarily represent those of their affiliated organizations, or those of the publisher, the editors and the reviewers. Any product that may be evaluated in this article, or claim that may be made by its manufacturer, is not guaranteed or endorsed by the publisher.

## Abbreviations

SGLT-2i: sodium–glucose cotransporter-2 inhibitor
DAPA: dapagliflozin
STZ: streptozotocin
CON: control
DM: diabetes mellitus
TLR4: toll-like receptor 4
IRAK1: interleukin receptor-associated kinase 1
TRAF6: tumor necrosis factor receptor-associated factor 6
NF-κB: nuclear factor-kappa B
AF: atrial fibrillation
HFrEF: heart failure with reduced ejection fraction
T2DM: type 2 diabetes mellitus
FS: fractional shortening
EF: ejection fraction
BCA: bicinchoninic acid
CV: conduction velocity
SCL: sinus cycle length
WCL: Wenckebach cycle length
SNRT: sinus node recovery time
ERP: effective refractory period
LVEF: left ventricular ejection fraction
LVFS: left ventricular fractional shortening
HW/BW: heart weight to body weight
HW/TL: heart weight to tibia length
SBP: systolic blood pressure
DBP: diastolic blood pressure
LAD: anteroposterior diameter of left atrium
PAT: pulmonary artery acceleration time
IVS: interventricular septum
LVID;d: left ventricular internal diameter at diastolic period
LVID;s: left ventricular internal diameter at systolic period
LVPW: left ventricular posterior wall
H&E: hematoxylin and eosin
ROS: reactive oxygen species
APD: action potential duration
ZDF: Zucker diabetic fatty
HSP60: heat shock protein 60
I/R: ischemia/reperfusion
ATP: adenosine 5'-triphosphate
EPO: erythropoietin
NLRP3: NOD-like receptor 3
GSDMD: gasdermin-D-N
AMPK: adenosine mono-phosphate kinase
CACNA1C: voltage-dependent L-type calcium channel
NCX: sodium–calcium exchanger
NHE1: sodium–hydrogen exchanger 1
NHE: sodium-hydrogen exchanger
MMPs: metalloproteinases
TIMPs: tissue inhibitors of metalloproteinases
AT: adipose tissue
ICIs: immune checkpoint inhibitors
DOXO: doxorubicin
NOXs: nicotinamide adenine dinucleotide phosphate oxidase
TGF-β1: transforming growth factor-β1
βOHB: β-hydroxybutyrate, d-β-hydroxybutyrate
HDACs: histone deacetylases.
